# Identification of Oxidative Stress-Related Biomarkers in Diabetic Kidney Disease

**DOI:** 10.1155/2022/1067504

**Published:** 2022-12-31

**Authors:** Xiaoju Ma, Xiaobo Zhang, Tian Leng, Jingru Ma, Zhongzhu Yuan, Yalin Gu, Tingting Hu, Qiuyan Liu, Tao Shen

**Affiliations:** ^1^School of Basic Medicine, Chengdu University of Traditional Chinese Medicine, Chengdu 611137, China; ^2^School of Public Health, Chengdu University of Traditional Chinese Medicine, Chengdu 611137, China

## Abstract

**Background:**

Diabetic kidney disease (DKD) is a leading cause of end-stage renal disease throughout the world. In kidney disease, oxidative stress has been linked to both antioxidant depletions and increased reactive oxygen species (ROS) production. Thus, the objective of this study was to identify biomarkers related to oxidative stress in DKD.

**Methods:**

The gene expression profile of the DKD was extracted from the Gene Expression Omnibus (GEO) database. The identification of the differentially expressed genes (DEGs) was performed using the “limma” *R* package, and weighted gene coexpression network analysis (WGCNA) was used to find the gene modules that were most related to DKD. Gene ontology (GO) and Kyoto Encyclopedia of Genes and Genomes (KEGG) pathway enrichment analysis was performed using “Org.Hs.eg.db” *R* package. The protein-protein interaction (PPI) network was constructed using the STRING database. The hub genes were identified by the Molecular Complex Detection (MCODE) plug-in of Cytoscape software. The diagnostic capacity of hub genes was verified using the receiver operating characteristic (ROC) curve. Correlations between diagnostic genes were analyzed using the “corrplot” package. In addition, the miRNA gene transcription factor (TF) network was used to explain the regulatory mechanism of hub genes in DKD.

**Results:**

DEGs analysis and WGCNA-identified 160 key genes were identified in DKD patients. Among them, nine oxidative stress-related genes were identified as candidate hub genes for DKD. Using the PPI network, five hub genes, NR4A2, DUSP1, FOS, JUN, and PTGS2, were subsequently identified. All the hub genes were downregulated in DKD and had a high diagnostic value of DKD. The regulatory mechanism of hub genes was analyzed from the miRNA gene-TF network.

**Conclusion:**

Our study identified NR4A2, DUSP1, FOS, JUN, and PTGS2 as hub genes of DKD. These genes may serve as potential therapeutic targets for DKD patients.

## 1. Introduction

Diabetic kidney disease (DKD), one of the most prevalent and severe chronic microvascular complications of diabetes, is the leading cause of end-stage renal disease [1]. Approximately 40% of diabetes mellitus develop DKD [2]. Although the established control protocols of blood glucose and blood pressure have reduced burden of DKD, the incidence of DKD remained increasing [3]. Therefore, prevention of DKD is the major public health challenge. The novel strategies and techniques for the early diagnosis and risk prediction are required.

Oxidative stress has long been recognized as a crucial factor in the pathogenesis of DKD [1, 2]. Hyperglycaemia increases free radical production leading to oxidative stress which is an imbalance between oxidant and antioxidant levels [4, 5]. Oxidative stress stimulates signaling molecules such as nuclear factor-*κ*B (NF-*κ*B) or activator protein-1 (AP-1) [6], ultimately leading to inflammation, fibrosis, and apoptotic in renal cells [7, 8]. Thus, the key to preventing DKD progression is to eliminate oxidative stress-induced cell damage in the early stages of DKD [9]. The reliable oxidative stress biomarkers suitable for clinical practice associated with DKD are very important. This study aimed to identify potential biomarkers of oxidative stress by bioinformatic approaches to develop new strategies for early prevention and treatment of DKD in clinical research.

## 2. Materials and Methods

### 2.1. Data Source

GEO is a database containing high-throughput gene expression data, chips, and microarrays. The two DKD RNA-sequencing datasets, GSE142025 and GSE30528, were downloaded from the GEO database. GSE142025 dataset included 27 kidney biopsy samples from patients with DKD and nine nephrectomy samples from the normal kidney tissue. GSE30528 dataset had nine diabetic kidney disease glomeruli and 13 control glomeruli tissue samples. The GSE142025 dataset was used for the training, and the GSE30528 dataset was used for validation. The GO term “response to oxidative stress (GO: 0006979)” was used to identify genes related to oxidative stress.

### 2.2. Identification of DEGs

The expression matrices were divided into disease and control groups and were screened for DEGs. Identification of DEGs between DKD tissue samples and normal tissue samples was performed by using the “limma” *R* package. The cutoff criteria were as follows: |log_2_fold change (FC)| > 1 and adj. *p* value <0.05. DEGs meeting the criterion were selected for further analysis.

### 2.3. WGCNA for Building DKD-Associated Modules

The *R* package “WGCNA” [10] was used to identify DKD-related modules and genes. Hierarchical clustering of the gene expression in the samples was performed using the Euclidean distance to detect and eliminate outliers. Genes with similar expression patterns were assigned to a coexpression module. The pickSoftThreshold function of WGCNA was used to determine the best soft threshold power (*β*) based on values from 1 to 20.

### 2.4. Gene Set Enrichment Analysis (GSEA) of the Key DKD Genes

The key DKD genes were obtained by taking the intersection of DEGs and module genes. GSEA is a computational method for evaluating whether a set of a priori-defined show statistically significant and consistent differences between two biological states. The GSEA analysis was performed on the key genes of DKD. For GSEA analysis, “h.all.v7.5.1.symbols.gmt” was downloaded from the Molecular Signature Database (MSigDB) as the background gene set.

### 2.5. Functional and Pathway Enrichment Analyses of Candidate Hub Genes

The key DKD genes were annotated using the GO term “response to oxidative stress” to get the candidate hub genes. GO term analysis and KEGG pathway analysis were performed using the *R* package “Org.Hs.eg.db” [11] to identify the functional roles of the candidate hub genes. GO terms or KEGG pathways with adj. *p* value <0.05 were considered statistically significant.

### 2.6. Construction of the PPI Network and Identification of Hub Genes

We constructed a PPI network of candidate hub genes using the STRING database [12] to further screen meaningful hub genes. The hub genes were identified using MCODE [13], a Cytoscape plug-in.

### 2.7. Diagnostic Performance Evaluation of Hub Genes by ROC Analysis

The ROC curve was plotted, and AUC was calculated using the “pROC” *R* package [14] to evaluate the capability of selected hub genes and to distinguish DN patients and controls. Spearman correlation coefficients between genes were visualized using the “corrplot” *R* package [15].

### 2.8. Construction of the Regulatory Network

MiRNAs can reduce gene expression by binding mRNAs and thereby inducing gene silencing. Transcription factors (TFs), also known as trans-acting factors, are DNA-binding proteins that can activate or inhibit gene transcription by interacting with cis-acting elements of eukaryotic genes. The miRNet database, a network-based visual analysis tool [16], was used to predict miRNAs and TFs that interacted with the hub genes. The miRNA gene-TF network was constructed using Cytoscape software.

### 2.9. GEO and Nephroseq v5 Validation

The expression levels of hub genes associated with DKD were compared with controls using the GEO (GSE30528) and Nephroseq v5 databases in order to verify the robustness of our results in the external dataset.

## 3. Results

### 3.1. Identification of DEGs between DKD and Control Groups

**Figure 1:**
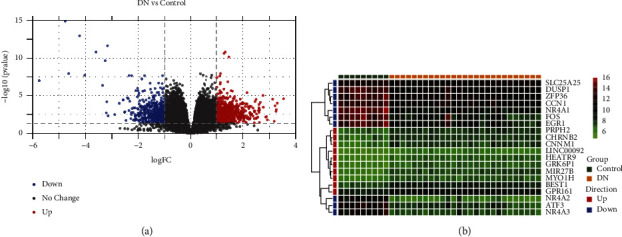
The differential expression genes identified by the “limma” *R* package between DKD tissue and normal tissue in the GSE142025. (a) Volcano plot for the differential expression analysis, blue dots are for downregulated and red for upregulated genes. (b) Heatmap for the top 10 upregulated genes and top 10 downregulated genes as sorted by the adjusted *p* value.

We compared DKD and normal tissues in the GSE142025 using the “limma” *R* package and identified genes with differential expression in this dataset. The data were filtered using |log_2_FC| > 1 and adj. *p* value <0.05. A total of 1,121 DEGs (DKD vs. control) were identified, including 641 upregulated genes and 480 downregulated genes (Figure 1(a)). The top 10 upregulated genes and top 10 downregulated genes (sorted by adj. *p* value) are shown in Figure 1(b).

### 3.2. Discovery of the DKD-Related Modules and Genes

**Figure 2:**
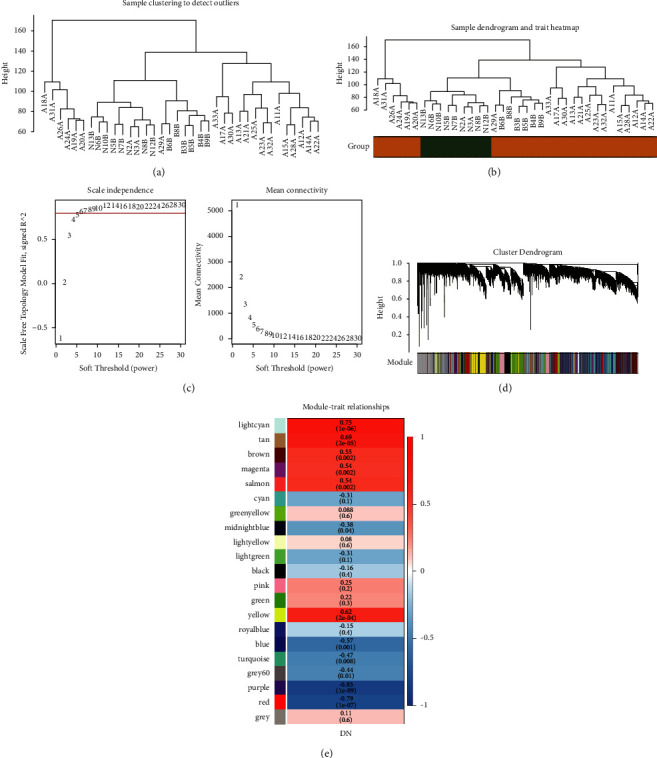
Construction of coexpression modules related to DKD based on WGCNA. (a) Sample clustering to detect outliers. (b) Sample clustering in the DKD tissue and normal tissue. (c) The appropriate soft threshold selection. (d) Coexpression genes expression pattern of 20 modules by WGCNA analysis. (e) Correlation between coexpression modules and DKD.

To identify genes associated with DKD, we performed WGCNA. First, the hierarchical clustering of the samples showed that there were no outliers (Figures 2(a) and 2(b)). Then, *β* = 6 was selected as the appropriate soft-thresholding power to ensure a scale-free analysis (Figure 2(c)). Highly related genes were divided into the same module, and a total of 20 modules were identified (Figure 2(d)). A heatmap was generated to show the global outline of the relationship between the modules and DKD (Figure 2(e)). According to the correlation results, a strong positive correlation was observed between the light cyan module (Cor = 0.75, *p* value = 1*E* − 06), tan module (Cor = 0.69, *p* value = 2*E* − 05), yellow module (Cor = 0.62, *p* value = 2*E* − 04), and DKD, and a strong negative correlation between purple module (Cor = −0.85, *p* value = 1*E* − 09), red (Cor = −0.79, *p* value = 1*E* − 07) module, and DKD. In addition, 3,238 genes within these coexpression modules were associated with DKD.

### 3.3. Screening and Analysis of Key Genes and Candidate Hub Genes of DKD

**Figure 3:**
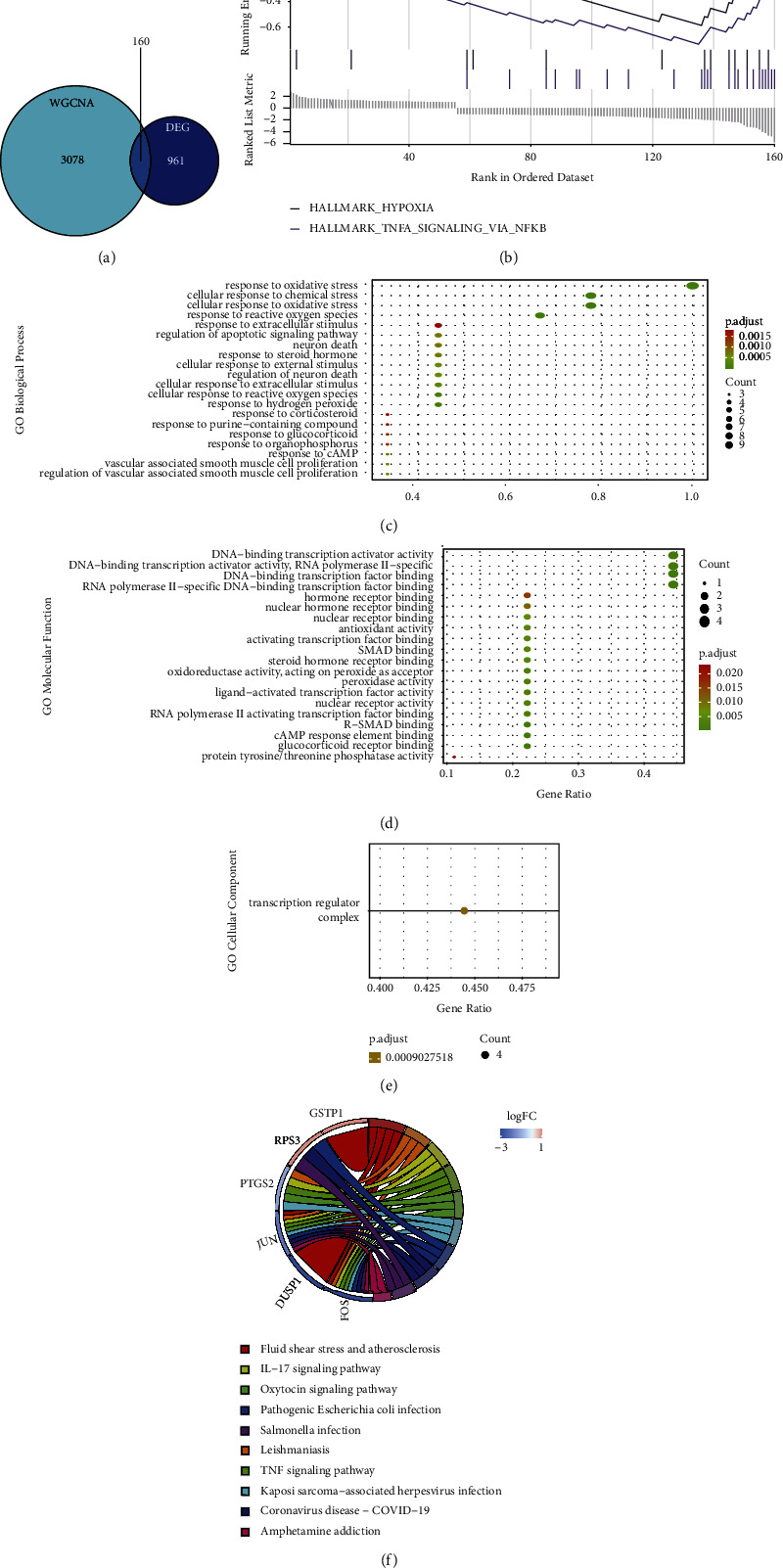
Screening and analysis of key genes and candidate hub genes of DKD. (a) Venn diagram for intersections between DEGs and module genes of WGCNA. (b) Genes set enrichment analysis of 160 key genes by the Hallmark gene database. (c)–(e) GO enrichment map in BP, CC, and MF. (f) KEGG enrichment map.

The selected DEGs and module genes were intersected, and a total of 160 overlapping genes were screened out as the key genes for DKD (Figure 3(a)). Then, the GSEA analysis was performed on these 160 key genes using the hallmark gene sets in the MSigDB database (Figure 3(b)). The results showed that two pathways were significantly enriched, namely “HALLMARK_HYPOXIA” and “HALLMARK_TNFA_SIGNALING_VIA_NFKB.” Then, a total of 9 out of 160 genes were annotated in the GO term “response to oxidative stress (GO: 0006979),” which were NR4A2, DUSP1, NR4A3, FOS, JUN, PTGS2, GSTP1, NOL3, and RPS3. These genes were identified as candidate hub genes for DKD. To explore the potential function of these candidate hub genes, we performed GO and KEGG enrichment analysis using the *R* package “Org.Hs.eg.db.” Figures 3(c)–3(e) show the candidate hub genes were mainly involved in “response to oxidative stress,” “cellular response to chemical stress,” and “cellular response to oxidative stress” for the aspects of biological process (BP), “transcription regulator complex” for the aspects of cellular component (CC), and “DNA-binding transcription activator activity,” “DNA-binding transcription activator activity, RNA polymerase II-specific,” and “DNA-binding transcription factor binding” for the aspects of molecular function (MF). KEGG pathway analysis indicated the candidate hub genes were mainly enriched in “fluid shear stress and atherosclerosis,” “leishmaniasis,” and “IL-17 signaling pathway” (Figure 3(f)).

### 3.4. Identification of Hub Genes by the PPI Network

**Figure 4:**
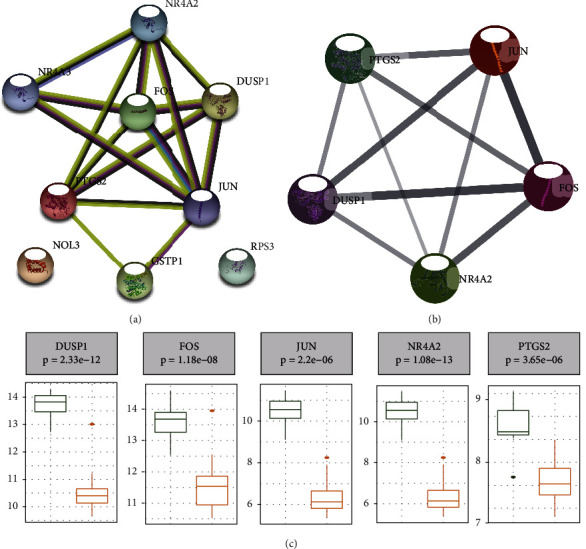
PPI network diagram of hub genes related oxidative stress. (a) PPI network diagram of 9 candidate hub genes. (b) PPI network diagram of 5 hub genes from core module analyzed using MCODE. (c) Expression levels of the five hub genes.

To better understand the interaction among the identified nine candidate hub genes, we used the STRING online server to construct a PPI network (Figure 4(a)). The entire PPI network was analyzed using MCODE, and the genes were selected as hub genes in the core module (Figure 4(b)). The five genes, NR4A2, DUSP1, FOS, JUN, and PTGS2, were in the core module and were selected as hub genes. The expression of all hub genes was downregulated in DKD patients (Figure 4(c)).

### 3.5. Evaluation of the Diagnostic Effect of the Hub Gene on DKD

**Figure 5:**
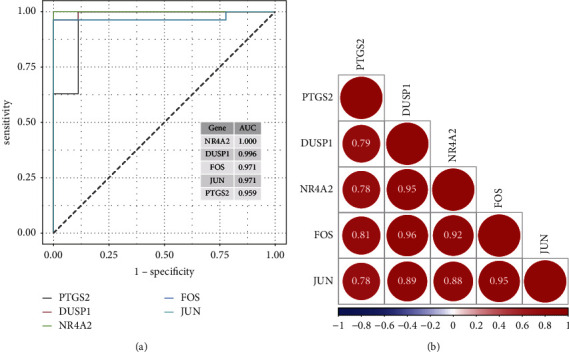
Evaluation of the diagnostic effect of the hub gene on DKD. (a) ROC curves and AUC values of the five hub genes. (b) Correlations among the five hub genes.

In this study, the ROC curve was plotted to evaluate the diagnostic ability of DKD for five hub genes and calculated the corresponding AUC values (Figure 5(a)). The results show that NR4A2 (AUC = 1), DUSP1 (AUC = 0.996), FOS (AUC = 0.971), JUN (AUC = 0.971), and PTGS2 (AUC = 0.959) can be utilized as distinguishing feature parameter for DKD. The correlations between five hub genes were then analyzed (Figure 5(b)). NR4A2, DUSP1, FOS, JUN, and PTGS2 were positively correlated with each other. The correlation between DUSP1 and FOS was the highest at 0.96.

### 3.6. Construction of the miRNA Gene-TF Regulatory Network

**Figure 6:**
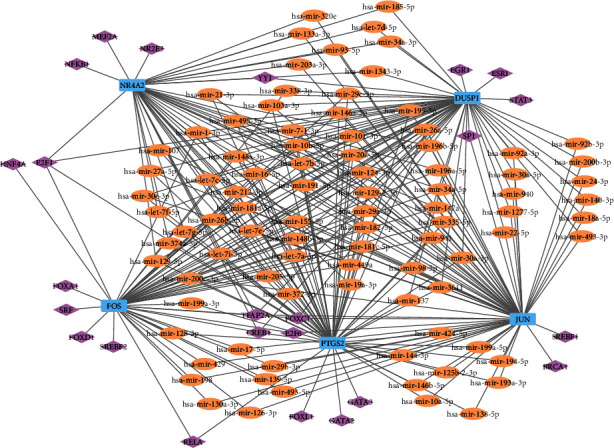
Construction of the miRNA gene-TF regulatory network. Light blue rectangle represents hub genes, orange oval represents miRNA, and purple diamond represents TF.

In this study, we constructed a miRNA gene-TF regulatory network by collecting and integrating the documented regulatory interactions between miRNAs/TFs and hub genes (Figure 6). A total of 121 nodes (5 hub genes, 91 miRNAs with degree ≥2, and 25 TFs) and 286 edges were included in the miRNA gene-TF network. The miRNAs that regulated the largest number of hub genes (5 genes) were hsa-mir-16-5p, hsa-mir-129-2-3p, hsa-mir-20a-5p, hsa-mir-124-3p, and hsa-mir-155-5p. CREB1, TFAP2A, and E2F1 regulating four genes ranked highest among the TFs. In addition, the hub genes JUN, PTGS2, DUSP1, FOS, and NR4A2 had the degree of 70, 63, 59, 52, and 42, respectively.

### 3.7. Expression Validation of Hub Genes

**Figure 7:**
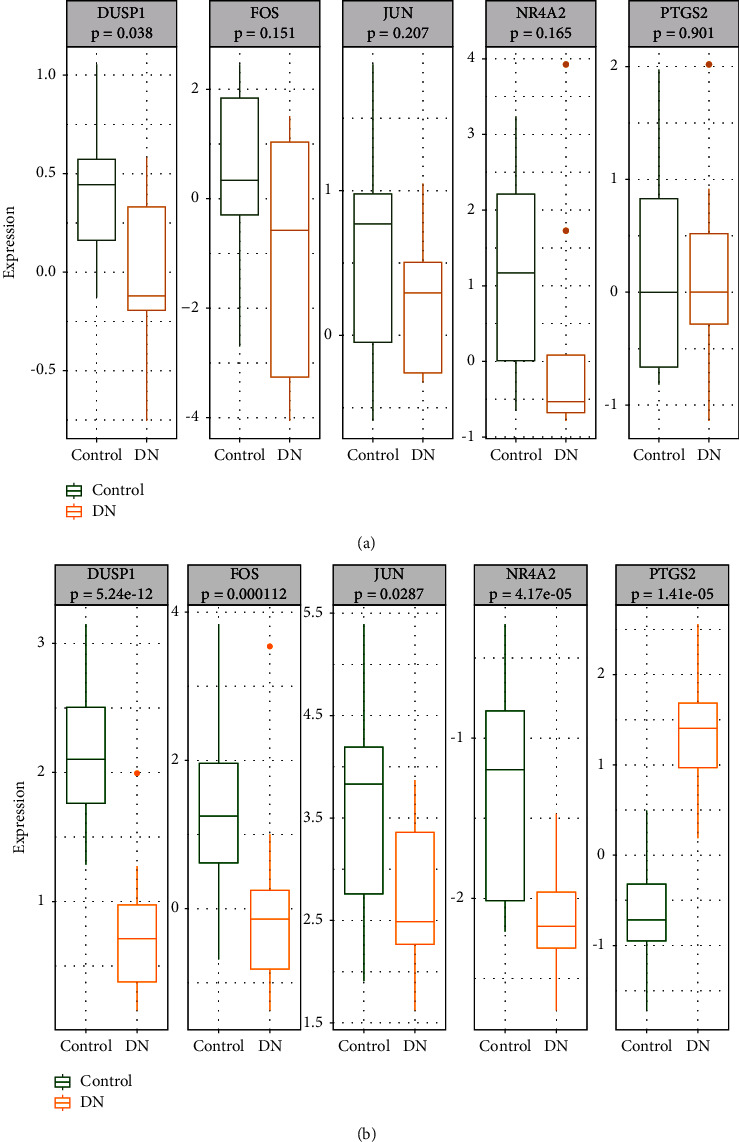
Expression validation of the hub gene in the training set.

The expressions of the hub genes were further verified in the validation GSE30528 dataset (Figure 7(a)). The results showed that the expression pattern of the five hub genes in the validation set was comparable to the training set. In the validation set, however, only a significant difference in the DUSP1 expression was observed between DKD and control samples. We further examined the expressions of the hub genes in DKD and control samples using the online tool Nephroseq V5 (Figure 7(b)). The result showed that hub genes NR4A2, DUSP1, FOS, and JUN had the same expression patterns as the training set.

## 4. Discussion

Oxidative stress, as an important factor in the development and progression of DKD, has been a research hotspot. The aim of this study was to investigate disease evaluation and diagnosis markers.

The nine screened candidate genes for oxidative stress were subjected to GO and KEGG enrichment analyses based on data acquisition from online databases. On the basis of response to oxidative stress, cellular response to chemical stress, cellular response to oxidative stress, and various other physiological processes, these genes could form a transcription regulator complex with other proteins to regulate the gene expression. Subsequently, this complex will help in regulating molecular functions such as DNA-binding transcription activator activity, RNA polymerase II-specific, and DNA-binding transcription factor binding. In this way, these genes would further regulate fluid shear stress and atherosclerosis signaling pathway, leishmaniosis signaling pathway, IL-17 signaling pathway, TNF signaling pathway, and many others. Consequently, these genes were involved in oxidative stress, inflammatory response, and cell apoptosis. Among them, the IL-17 signaling pathway and TNF signaling pathway are closely associated with the occurrence and development of diabetic nephropathy. IL-17 is a key cytokine secreted by CD4^+^*T* cells [17]. In the high glucose state, the accumulation of AGEs increases the expression of IL-17 [18, 19], and after binding to its receptor, it promotes IL-6, monocyte chemotactic protein (MCP-1), and regulates activation of monocytes. The production of the transforming growth factor TGF-*β*1 and the activated *B* cell nuclear factor kappa light chain enhancer (NF-*κ*B) mediate the inflammatory response associated with the tissue damage in DKD and glomerulosclerosis. However, it is important to note that other studies have come to the opposite conclusion. In a clinical study, IL-17 expression decreased in DKD patients with disease progression [20]. In addition, in the STZ-treated mouse model of IL-17 knockout, it was found that IL-17A knockout mice aggravated renal inflammation and fibrosis by inhibiting autophagy [21]. Although the specific role of IL-17 in diabetic nephropathy is still unknown, it is clear that IL-17 has a significant role in the onset of diabetic nephropathy. The TNF family is involved in different states of DKD by regulating immune function. In animal studies and clinical trials, a high glucose environment can induce oxidative stress, resulting in an increase in the expression of TNF-*α*, which can aggravate DKD injury [22–24]. Its mechanism is related to the induction of podocyte retinoic acid receptor responder protein 1 (RARRES1) expression to activate podocyte apoptosis [25], inhibition of NRF2/KEAP1/ARE pathway to reduce antioxidant capacity [26], to promote NF-*κ*B release [27] and other inflammatory factors release, and drive Egr-1 activation [28] to regulate cell differentiation and growth. Osteoprotegerin (OPG) may be among the most studied TNF family. OPG expression was found to be upregulated in the kidneys of diabetic patients and correlated with the severity of DKD [29]. These results show that the hub gene screened by our study is closely associated with the occurrence of DKD and also suggests a potential pathway, providing a new theoretical foundation for researchers into the mechanism of DKD.

For further exploration of the key genes affecting DKD, five hub genes (NR4A2, DUSP1, FOS, JUN, and PTGS2) were screened by the PPI network, all of which were underexpressed in DKD patients. Gene expression of NR4A2, DUSP1, FOS, JUN, and PTGS2 was negatively correlated with DKD. In addition, all these five hub genes showed strong diagnostic ability based on their diagnostic performance by the ROC curve.

NR4A2 is a member of the superfamily of steroid-thyroid hormone-retinoid receptors. Under conditions of cellular response to external stimuli, the genes of this family would rapidly produce corresponding proteins, which are transcription factors to regulate gene expression [30]. Cumulative evidence has revealed the association of NR4A2 with oxidative stress. For instance, Popichak KA reported that NR4A2 could inhibit the inflammatory response through NF-*κ*B [31, 32]. Kaoru found that NR4A2 could suppress LPS-stimulated monocytes/macrophages to induce TNF mRNA expression in a non-NF-*κ*B manner, which could protect islet cells by alleviating endoplasmic reticulum stress in islet *β* cells [33]. Additional in vivo experiments showed that NR4A2 was involved in stress response [34].

In addition, DUSP1 could be considered an inhibitor of MAPK activity because it dephosphorylates MAPK at both tyrosine and threonine residues intracellularly and negatively regulates the production of proinflammatory factors [35]. Ikuyo discovered in his research that the transcription of the DUSP1 gene was inhibited under oxidative stress [36]. Leblanc et al. [34] previously reported an increased number of activated P38MAPK in the glomeruli of DKD, which can be explained by the downregulation of DUSP1 and is consistent with our results. In this regard, inducing the expression of DUSP1 could serve as a potential treatment for DKD.

AP-1 complex, composed of FOS and JUN, is an important transcription factor in the nucleus [37]. It has a regulatory role in biological processes such as cell proliferation, differentiation, and apoptosis [38]. Existing studies support its involvement in the occurrence and development of various diseases, such as colorectal cancer [39] and pancreatic cancer [40]. Under the high glucose environment, the production of ROS can induce increased FOS and JUN protein expression in the renal tissue, leading to the activation of AP-1 to initiate the transcription of its downstream genes (TGF-1, FN, and Laminin B). It may also lead to the accumulation of extracellular matrix and the thickening of the glomerular basement membrane in the renal tissue, thereby promoting the occurrence and development of DKD [37, 41, 42]. At the same time, there may be a change in the expression of FOS. Duan found an increased level of FOS in the proximal renal tubules in the event of acute renal injury, which was, however, highly unstable and degraded rapidly [43]. The existence of TRE inhibits its transcription on the promoter of the FOS gene; however, high glucose may cause the increase of FOS protein and promote the binding of AP-1 to TRE (specific TPA-responsive element), resulting in feedback inhibition of its own expression.

PTGS2 plays an essential role in the process of oxidative stress [44], and COX-2, encoded by PTGS2, is a key regulator of renal hemodynamics and promoter of inflammation, which also has a role in podocyte injury. It has been documented that the expression of PTGS2 in podocytes increased significantly under high glucose conditions [45]. Luo et al. reported an increase in the expression of COX-2 in insulin-dependent diabetes mellitus. In addition, Jia et al. revealed that the expression of COX-2 was increased in the renal tissue when there was renal injury [46]. While these findings are inconsistent with the decrease of PTGS2 gene expression to some extent in DKD patients observed in our study, however, further experimental validation is required.

In conclusion, our study suggests a close relationship between oxidative stress and DKD based on function and expression analysis of the screened oxidative stress-related genes in DKD. Oxidative stress-related genes may be considered novel biomarkers for the diagnosis and prognosis of DKD progression. Our study's findings may serve as a basis for future experimental confirmation, as well as research into disease pathogenesis and clinical treatment. However, our research also has some limitations. For example, we need more samples from multiple centers to verify the role of these hub genes in DKD. In addition, we need to further find the regulatory pathways of these hub genes in DKD through sequencing and other means, which is of great significance for a deeper understanding of the mechanism of hub genes in DKD.

## Data Availability

The datasets for this study are available in the GEO database. GEO belongs to public databases. Users can download relevant data for free for research and publish relevant articles.
